# Mesenchymal stromal cells and platelet-rich plasma promote tendon allograft healing in ovine anterior cruciate ligament reconstruction

**DOI:** 10.1007/s00167-020-06392-9

**Published:** 2020-12-17

**Authors:** Adam T. Hexter, Anita Sanghani-Kerai, Nima Heidari, Deepak M. Kalaskar, Ashleigh Boyd, Catherine Pendegrass, Scott A. Rodeo, Fares S. Haddad, Gordon W. Blunn

**Affiliations:** 1 Division of Surgery and Interventional Science, University College London (UCL), London, UK; 2 Royal London Hospital and Orthopaedic Specialists (OS), London, UK; 3 Hospital of Special Surgery (HSS), New York, USA; 4 University College London Hospitals, London, UK; 5 University of Portsmouth, Portsmouth, UK; 6 Institute of Orthopaedics and Musculoskeletal Sciences, Division of Surgery and Interventional Science, Royal National Orthopaedic Hospital, Brockley Hill, Stanmore, London, HA7 4LP UK

**Keywords:** Anterior cruciate ligament (ACL) reconstruction, Magnetic resonance imaging (MRI), Autopsy, Bone marrow-derived mesenchymal stromal cells (BMSCs), Platelet-rich plasma (PRP), Biological modulation

## Abstract

**Purpose:**

The effect of bone marrow mesenchymal stromal cells (BMSCs) and platelet-rich plasma (PRP) on tendon allograft maturation in a large animal anterior cruciate ligament (ACL) reconstruction model was reported for the first time. It was hypothesised that compared with non-augmented ACL reconstruction, BMSCs and PRP would enhance graft maturation after 12 weeks and this would be detected using magnetic resonance imaging (MRI).

**Methods:**

Fifteen sheep underwent unilateral tendon allograft ACL reconstruction using aperture fixation and were randomised into three groups (*n* = 5). Group 1 received 10 million allogeneic BMSCs in 2 ml fibrin sealant; Group 2 received 12 ml PRP in a plasma clot injected into the graft and bone tunnels; and Group 3 (control) received no adjunctive treatment. At autopsy at 12 weeks, a graft maturation score was determined by the sum for graft integrity, synovial coverage and vascularisation, graft thickness and apparent tension, and synovial sealing at tunnel apertures. MRI analysis (*n* = 2 animals per group) of the signal–noise quotient (SNQ) and fibrous interzone (FIZ) was used to evaluate intra-articular graft maturation and tendon–bone healing, respectively. Spearman’s rank correlation coefficient (*r*) of SNQ, autopsy graft maturation score and bone tunnel diameter were analysed.

**Results:**

The BMSC group (*p* = 0.01) and PRP group (*p* = 0.03) had a significantly higher graft maturation score compared with the control group. The BMSC group scored significantly higher for synovial sealing at tunnel apertures (*p* = 0.03) compared with the control group. The graft maturation score at autopsy significantly correlated with the SNQ (*r* = − 0.83, *p* < 0.01). The tunnel diameter of the femoral tunnel at the aperture (*r* = 0.883, *p* = 0.03) and mid-portion (*r* = 0.941, *p* = 0.02) positively correlated with the SNQ.

**Conclusions:**

BMSCs and PRP significantly enhanced graft maturation, which indicates that orthobiologics can accelerate the biologic events in tendon allograft incorporation. Femoral tunnel expansion significantly correlated with inferior maturation of the intra-articular graft. The clinical relevance of this study is that BMSCs and PRP enhance allograft healing in a translational model, and biological modulation of graft healing can be evaluated non-invasively using MRI.

## Introduction

Graft healing after ACL reconstruction consists of tendon–bone healing and matrix remodelling (“ligamentisation”) of the intra-articular graft [13, 15]. Graft remodeling can be measured non-invasively using MRI by measuring signal intensity of the intra-articular graft (signal–noise quotient, SNQ) [7]. Similarly, healing at the tendon–bone interface can be evaluated at fibrous interzone (FIZ) on MRI [13]. Bone tunnel widening is a concern [27] and allografts might be associated with increased tunnel widening [3].

There is a growing body of literature exploring biological modulation of graft healing [6]. Mesenchymal stromal cells (MSCs) can enhance tissue regeneration by differentiation, paracrine effects, or via immunomodulatory activity [1]. Platelet-rich plasma (PRP) is a blood derivative that can deliver supraphysiologic doses of cytokines and growth factors [17]. Bone marrow mesenchymal stromal cells (BMSCs) [12, 22] and PRP [24] have shown positive effects on graft healing in small animals. Evaluation in a large animal is an important translational step because it permits human-sized grafts and fixation systems to be used [16].

The clinical relevance of this study is that it represents the first large animal ACL reconstruction study to report the effects of BMSCs and PRP on allograft healing. The purpose of this study was to compare the effect of BMSCs and PRP on tendon allograft maturation in a large animal model, and to determine if MRI can be used to identify biological modulation of graft healing. The hypothesis was that BMSCs and PRP would enhance graft maturation after 12 weeks, and graft maturation at autopsy would correlate with graft maturation on MRI.

## Materials and methods

The research was conducted in accordance with a Project License protocol accepted under the UK Home Office Animals (Scientific Procedures) Act 1986 (licence number PF16F4AA0A). This study was approved by the animal warfare review board at the Royal Veterinary College. Fifteen full-mouthed female lowland Mule sheep (age, 2–3 years; weight, 60–75 kg) were included. Animals were randomised into three groups (*n* = 5 animals per group). In group 1, 10 million allogeneic BMSCs in fibrin sealant (Baxter, Vienna, Austria) were applied to the graft (2 million) and bone tunnels (4 million per tunnel). In group 2, 12 ml of PRP was injected into the graft (4 ml) and the bone tunnels (4 ml per tunnel). The control animals in Group 3 received no treatment. Post-operatively animals were euthanised after 12 weeks. Graft maturation was examined using MRI and a macroscopic scoring system at autopsy.

### PRP preparation

**Fig. 1:**
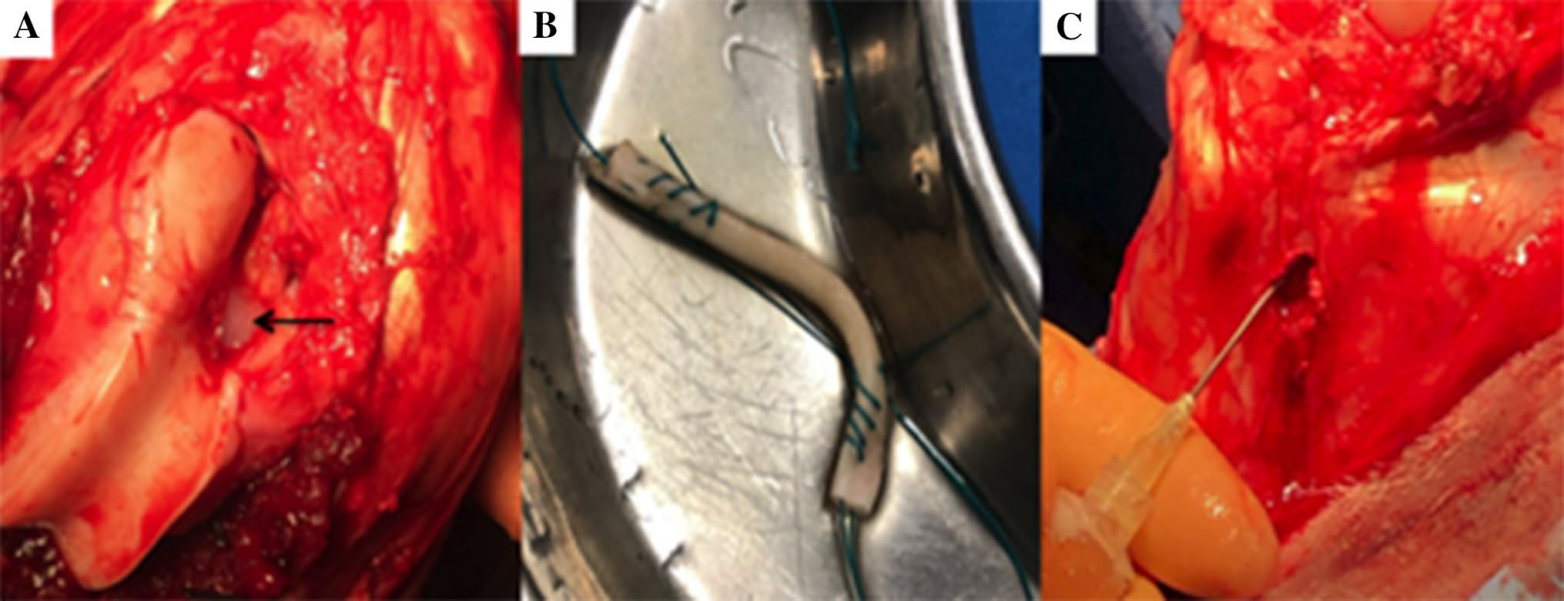
**a** Fibrin sealant at femoral aperture (arrow). **b** SDFT allograft soaking in PRP. **c** Intra-osseous injection of PRP into tibia

Autologous PRP was prepared using the *Endoret*^*®*^(*prgf*^*®*^) Technology (BTI System IV/V; BTI Biotechnology Institute, Vitoria, Spain). 72 ml of venous blood was obtained in 9 ml tubes containing 3.8% (wt/vol) sodium citrate. The tubes were centrifuged twice for 8 min at 580 G (1902 rpm) at room temperature. The 2 ml of plasma located above the buffy coat was collected, with a total PRP volume of 16 ml per animal (Fig. 1a, b). The PRP was activated by adding calcium chloride (10% wt/vol), which led to gel-like transformation within 5 min. The time between venipuncture and surgical delivery was 30 min. In sheep, this technique has been shown to yield PRP enriched in platelets and reduced in leucocytes [21].

### BMSC harvest

Bone marrow was aspirated from an adult sheep (age 2 years). 10 ml of bone marrow was aspirated from two regions of the iliac crest into a 20 ml polypropylene tube containing 10,000 International Units (IU) heparin sodium (Wockhardt UK Ltd, Wrexham, UK). The aspirate was combined with standard growth media, which was high glucose Dulbecco’s modified Eagle’s medium (DMEM) (Invitrogen, Paisley, UK), 10% FCS (ThermoFisher, Hemel Hempstead, UK) and 1% penicillin–streptomycin (ThermoFisher). The aspirate was plated onto T-150 culture flasks and cultured at 37 °C in a 5% carbon dioxide (CO_2_) incubator. After 72 h, the media was discarded, and fresh growth media was added to supplement the cells that had attached to the flask. The cells were detached with 0.25% trypsin–EDTA (Gibco, Carlsbad, CA) when they had reached 90% confluence. The BMSCs were sub-cultured and the growth media was changed every 3 days. 10 million, passage 3–4 cells were used in each surgical procedure. Tri-lineage potential—adipogenesis, osteogenesis, and chondrogenesis—of the cells was confirmed in vitro prior to surgery.

### Superficial digital flexor tendon (SDFT) allograft preparation

SDFT were harvested from sheep (age 2–3 years) using a previously described technique [8]. The grafts measured 75–85 mm in length and 7.5–8 mm in diameter and were sterilised by gamma irradiation at a dose of 25 kGy (Isotron, Reading, UK). The samples were stored at – 20 °C and thawed at room temperature 30 min before use. The ends of the graft were prepared with a whipstitch using no. 2 Ethibond (Ethicon Inc, Johnson & Johnson, New Jersey).

### Surgical technique

The right stifle joint was exposed via a medial arthrotomy and the fat pad and ACL were excised. Femoral and tibial bone tunnels (7.8 mm diameter) were drilled from the ACL footprints through the lateral femoral condyle and anteromedial tibia, respectively. Femoral fixation was achieved using an 8 × 20 mm Biosure (Polyether ether ketone, PEEK) interference screw (Smith & Nephew Endoscopy, Andover, MA)*.* The stifle joint was taken through ten full ranges of motion. Tibial fixation was achieved with the stifle joint in full extension with an 8 × 25 mm Biosure PEEK interference screw (Smith & Nephew Endoscopy, Andover, MA). A tension of 40 N was applied because this tension has been used in previous ovine studies [10, 11, 30]. In Group 1, 1 h before surgery, the BMSCs were loaded into 2 ml pre-filled Two-Component Fibrin Sealant (Baxter, Vienna, Austria). 0.8 ml of fibrin sealant (4 million BMSCs) was added to each bone tunnel and 0.4 ml of fibrin sealant (2 million BMSCs) covered the intra-articular graft (Fig. 1a). In group 2, the graft was infiltrated with 4 ml of activated PRP and left soaking in activated PRP liquid until implantation (Fig. 1b). The interference screws were immersed in 4 ml of activated PRP until implantation. Before graft insertion, the bone in the tunnels was infiltrated with 1 ml PRP at four intervals along the tunnel wall. In total, 4 ml was injected in the femoral tunnel and 4 ml in the tibial tunnel (Fig. 1c). In the control group, the procedure was performed without application of fibrin sealant, BMSCs or PRP. Joint stability was confirmed using an anterior drawer test and the incision was closed in layers. The animals freely mobilised in individual pens for 7 days and thereafter were house as a flock.

### Autopsy assessment

After euthanasia, the joint stability was checked using an anterior drawer test. The hind limb was dissected to examine graft maturation. A scoring system was devised based on parameters used at second-look arthroscopy [9]. A category for synovial sealing at the tunnel apertures was added based on a previously reported scoring system [25]. The scoring system consisted of four main criteria: graft integrity, synovial coverage and vascularisation, graft thickness and apparent tension, and synovial sealing at the tunnel apertures (Table 1). For graft integrity, a complete rupture was defined as complete loss of continuity of all fibres, a “partial rupture” was defined as a loss of continuity of some fibres, and “no rupture” was defined as no loss of fibre continuity. Synovial coverage and vascularisation were assessed by the percentage surface area of the intra-articular graft covered by vascularised synovium. For graft thickness and apparent tension, the highest score was for a thick graft (similar to the ovine ACL) which was not elongated. The second highest mark was for a graft that was either a similar thickness to the ACL and elongated, or not elongated but the graft itself was thin relative to the ACL. The lowest mark was for elongated and thin grafts. For synovial sealing at the tunnel apertures, the highest mark was for circumferential sealing at the aperture (> 75% circumference), the second highest mark was for a partial sealing (25–75% circumference) and the lowest mark was for low sealing (< 25% circumference). The overall graft maturation score was calculated from the sum of the separate scores (range 4.0–9.0). Two independent researchers blinded to the treatment group scored the grafts.

**Table 1:** Criteria for the Autopsy Graft Maturation Score (out of 9)

Score	Graft integrity	Synovial coverage and vascularisation	Graft thickness and apparent tension	Synovial sealing at tunnel apertures
3.0	–	≥ 75% with abundant vascularisation	–	–
2.0	No rupture	≥ 75% without vascularisation	No elongation of a sufficiently thick graft	Circumferential sealing (> 75%)
1.0	Partial rupture	25–74%	Partial elongation of a sufficiently thick graft or no elongation of a relatively thin graft	Moderate synovial sealing (25–75%)
0	Complete rupture	≤ 25%	Obvious elongation of a thin graft	Low synovial sealing (< 25%)

### MRI assessment

Two animals per group were randomly selected to have MRI scans after post-mortem but before autopsy. The MRI scans were performed using a superconducting 1.5T magnet (Intera Pulsar System, Philips Medical Systems, UK). The stifles were positioned in lateral recumbency with the joint at 90 degrees of flexion. Sequences performed included three-dimensional T1 weighted FFE (T1 3D FFE) imaging and fat-saturated proton density (PD-SPIR). Two blinded veterinary radiologists assessed the scans independently using a PACS workstation DICOM viewer (Osirix Imaging Software, version 3.9.2, Bernex, Switzerland).

**Fig. 2:**
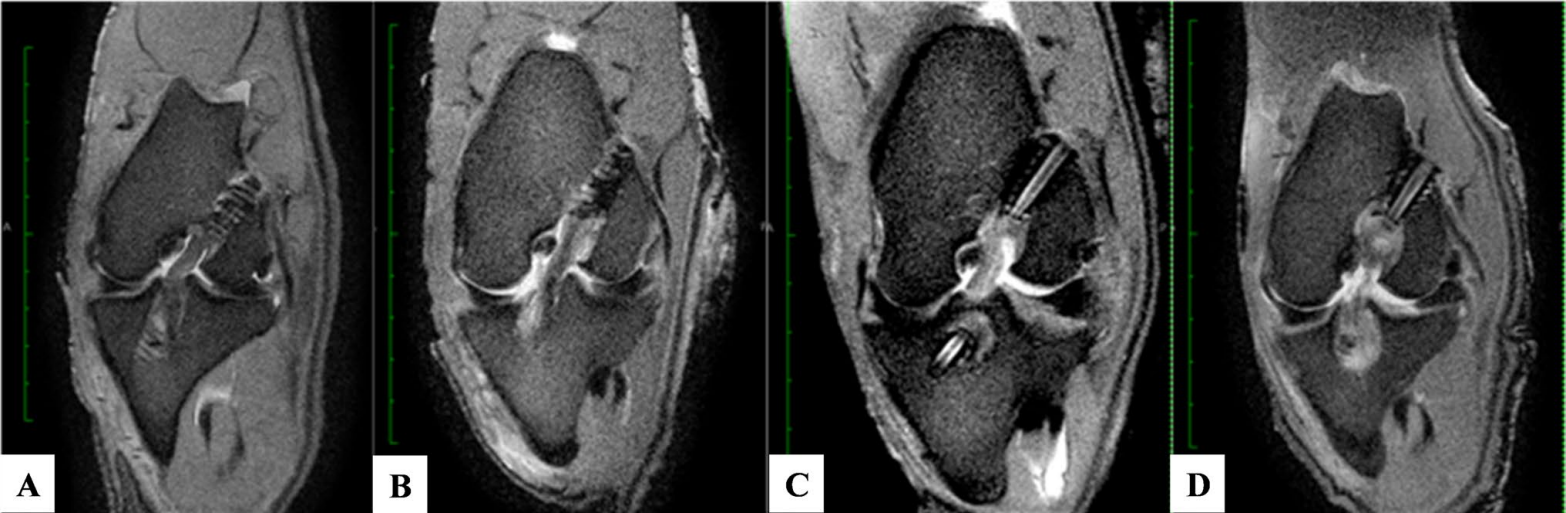
Scoring grades for intra-articular graft maturity described by Howell et al. [7]. **a** Grade 1 **b** Grade 2 **c** Grade 3 **d** Grade 4

Radiological assessment of the intra-articular graft was via a scoring system based on the score reported by Howell et al. [7] (Fig. 2) and the signal-to-noise quotient (SNQ). Grade 1 consisted of a graft of homogeneously low signal intensity which was intermediate between the intensity of skeletal muscle and the posterior cruciate ligament (PCL). Grade 2 consisted of a graft of homogeneously low signal intensity in greater than 50% of the graft, with the remainder of increased signal intensity. Grade 3 was a graft of homogeneously low signal intensity in less than 50% of the graft with the remainder of increased signal intensity. Grade 4 consisted of a graft of diffusely increased signal intensity. A low graft signal denotes superior graft maturation and, therefore, a lower score denotes better graft healing.

**Fig. 3:**
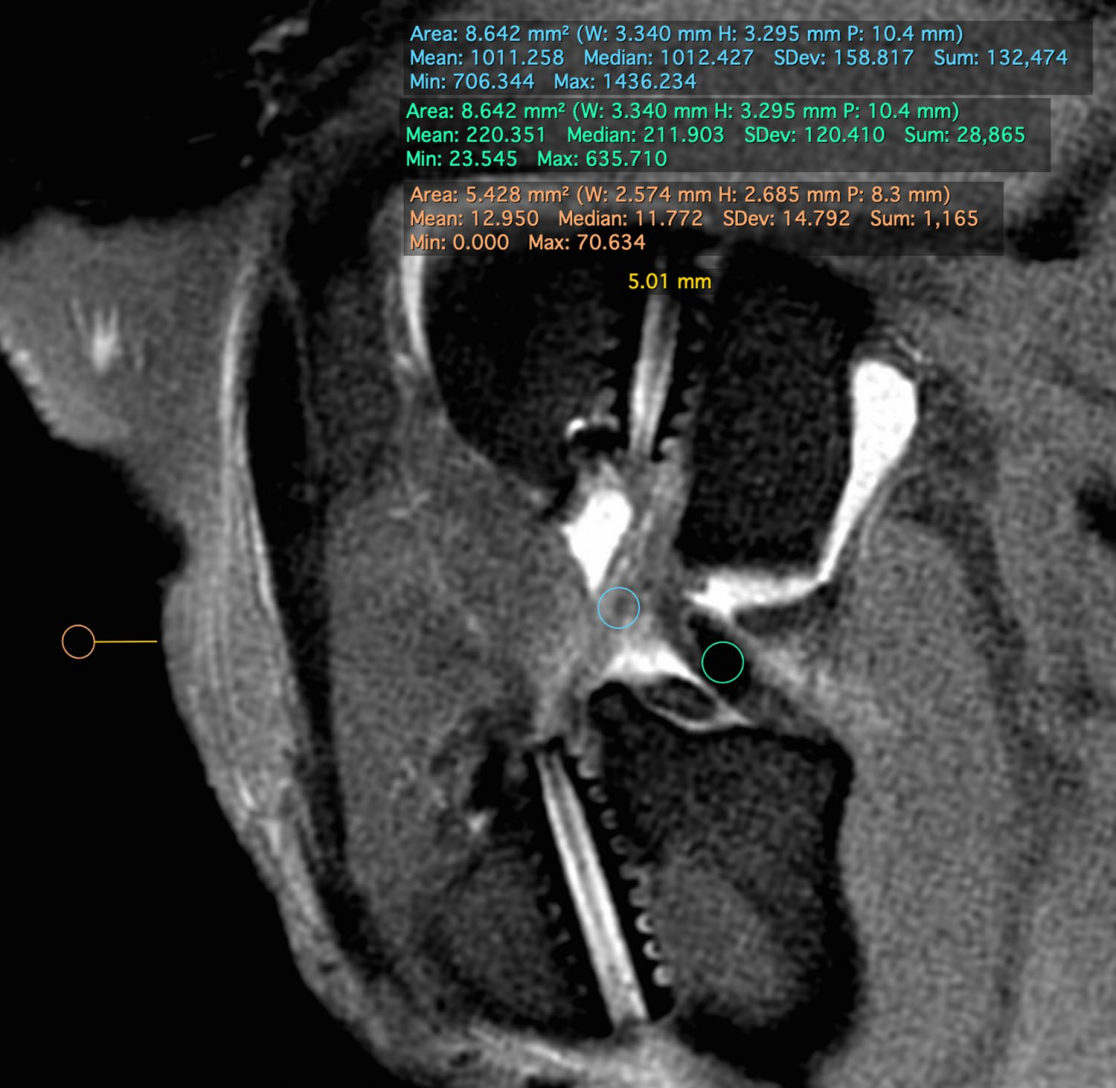
Regions of interest (ROIs) for SNQ Calculation. Blue shows the graft ROI; green circle shows the PCL ROI; and orange shows the background signal

Measurement of the SNQ was performed using a sagittal oblique PD-STIR image. A region of interest (ROI) was placed within the intra-articular graft and within the PCL. For measurement of background signal intensity, a 5 mm^2^ circular ROI was placed 5 mm cranial to the skin edge and the mean signal intensity was noted (Fig. 3). The SNQ was measured as follows: SNQ = (mean graft signal intensity—mean PCL signal intensity)/mean background signal intensity. A lower SNQ denotes more advanced graft maturation.

**Fig. 4:**
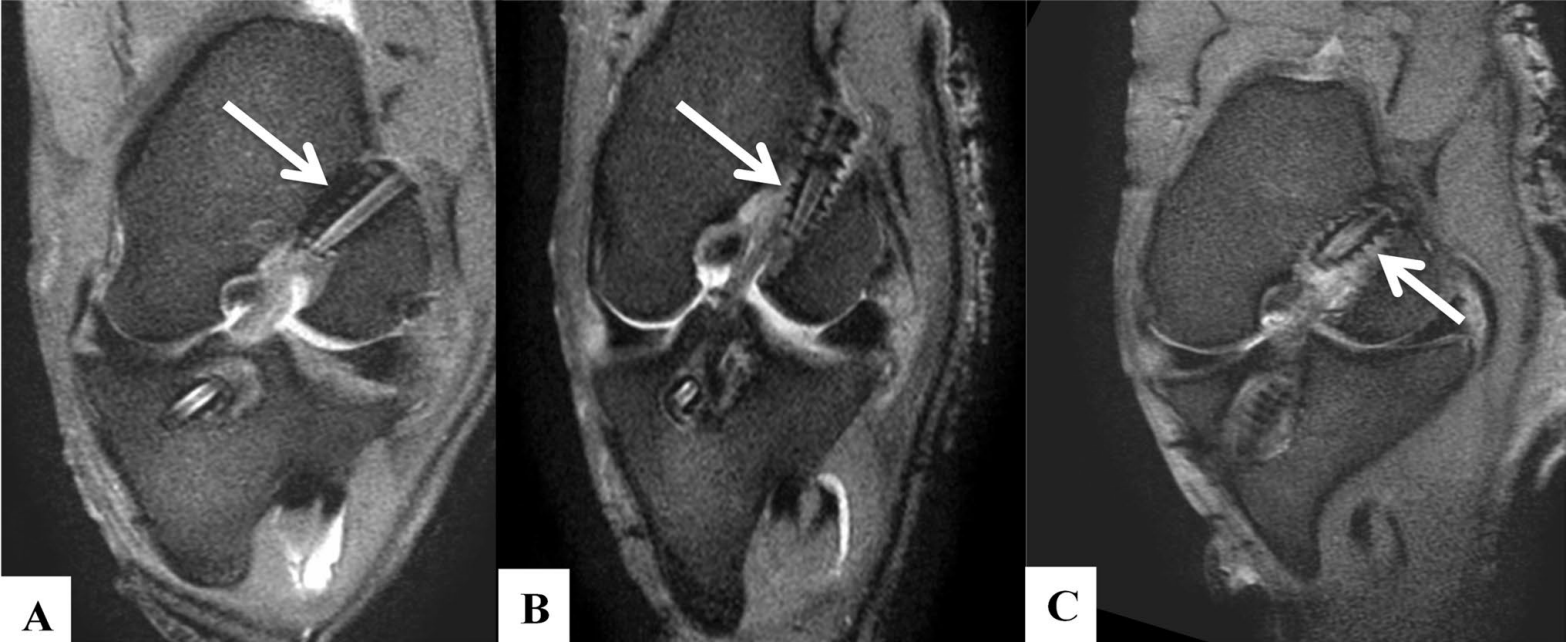
Scoring grades for tendon–bone healing using femoral tunnel as an example. **a** Grade 1 **b** Grade 2 **c** Grade 3

Graft width was measured on PD-SPIR images on coronal and sagittal images and the average taken. Bone tunnel width was measured using axial slices at the aperture, mid-portion and exit. The precision of measurements was 0.01 mm. Tunnel widening was calculated by subtracting the original drill diameter (7.8 mm) from the diameter measured after 12 weeks. Radiological assessment of the tendon–bone healing at the fibrous interzone was measured using a peer-reviewed three-grade scoring system (Fig. 4) [5]. Grade 1 consisted of a low-intensity signal band in the bone tunnel with no hyper-intense tissue at the tendon–bone interface. Grade 2 consisted of a low-intensity signal band with a partial high-intensity signal band at the interface. In grade 3, the interface was filled with a continuous high-intensity signal band.

### Statistical analysis

IBM SPSS Statistics for Windows, version 26 (IBM Corp., Armonk, N.Y., USA) was used. Data was not normally distributed and reported as medians and interquartile ranges. Mann–Whitney *U* tests were used to compare treatment groups with the control. Inter-rater reliability as assessed using Kappa statistics. The interpretation was as follows: < 0.40 was poor, 0.40–0.59 was fair, 0.60–0.74 was good, and ≥ 0.74 was excellent [4]. Correlations between the SNQ, maturation scores and tunnel diameters were examined using Spearman’s rank correlation coefficient (*r*). Significance was assumed at the 0.05 level. Previous animal studies of ACL reconstruction similar to this have used a *n* = 5 [6]. Taking a difference in the median average of 15% and a standard deviation of 10% with a power of 0.8 and *p* value of 0.05, a *n* = 5 has been shown to produce significant results.

## Results

### Autopsy assessment

**Fig. 5:**
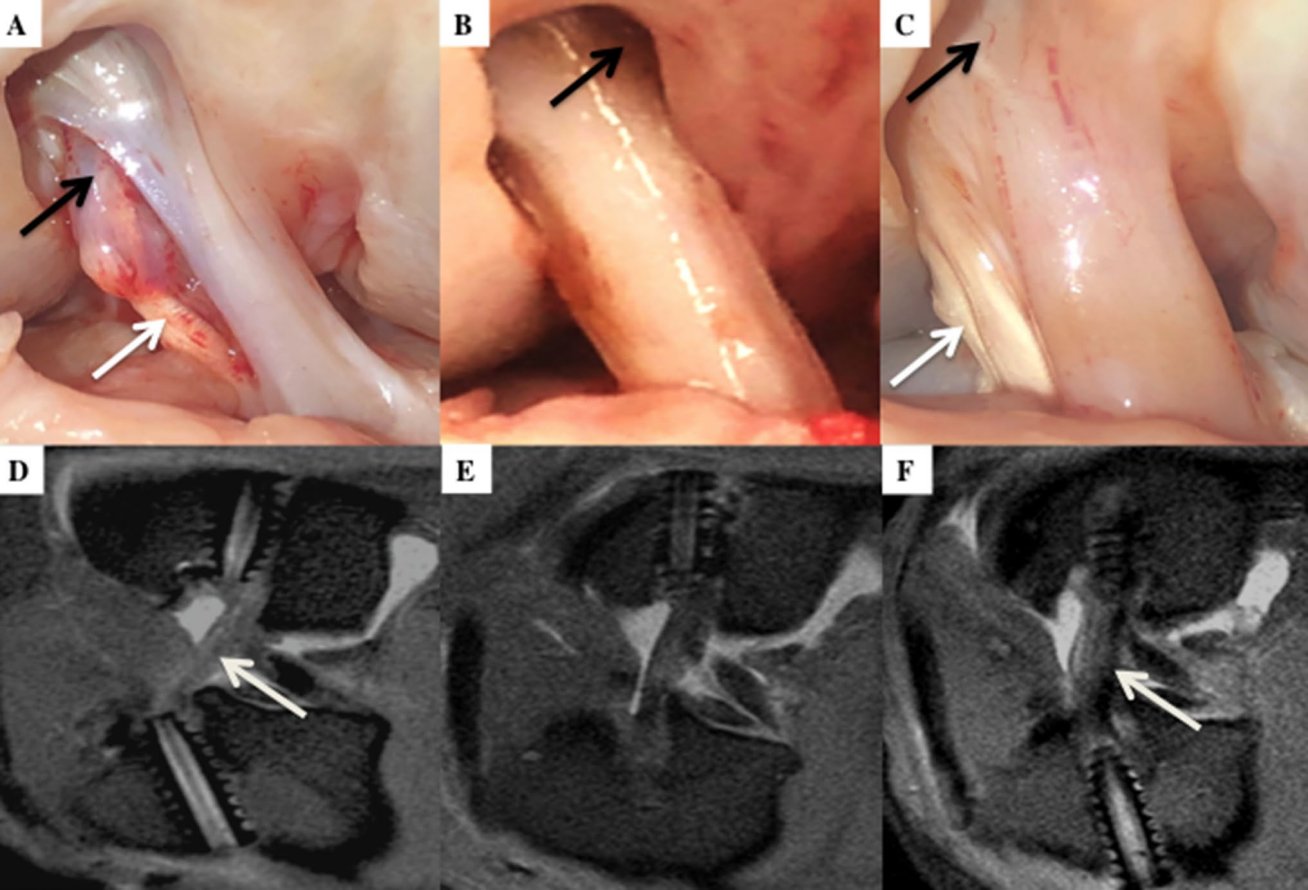
Autopsy photographs and corresponding sagittal MRI images of control (**a**, **d**), PRP (**b**, **e**), and BMSC (**c**, **f**) group. Autopsy scores, respectively, for graft integrity: synovial coverage: graft thickness/tension: incorporation at tunnel apertures. **a** = 2:2:0:1; **b** = 2:2:2:1; **c** = 2:3:2:2. In the control group inflammatory tissue is seen between split graft fibres (black arrow). In the PRP group the aperture is not sealed (black arrow) but the aperture is sealed in the BMSC group (black arrow). Regions of the graft appears to be more hypointense in the control and BMSC group (white arrow) but the graft is more homogenous hyperintense in the PRP group

There was no evidence of joint instability or an adverse effect to the treatments. Minimal chondral degeneration and mild synovial inflammation was observed. All the grafts were intact at the time of dissection. The PRP and BMSC treated grafts had remodelled into ACL-like structures. In contrast, grafts from the control group appeared atrophic with minimal vascularisation (Fig. 5).

**Fig. 6:**
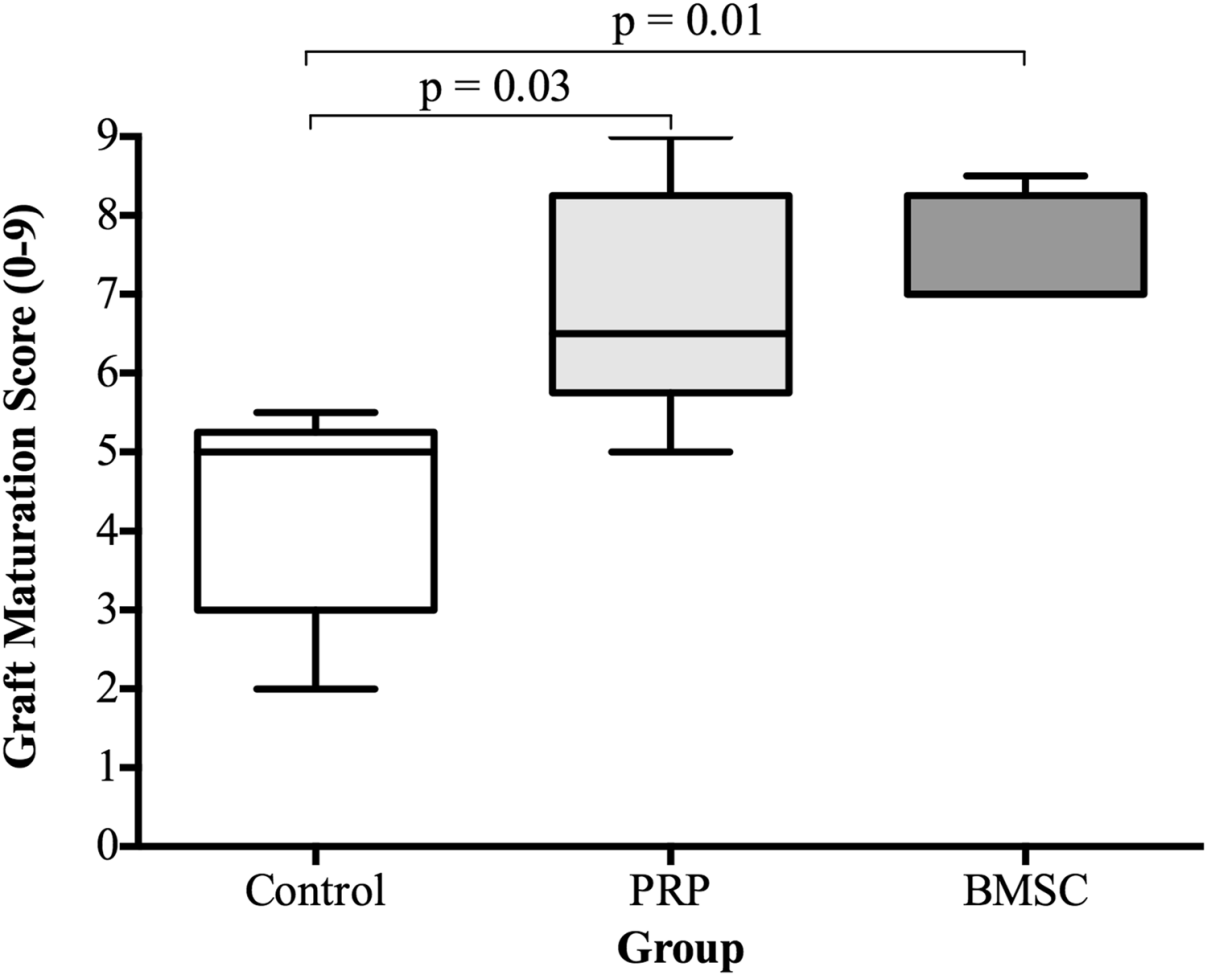
A box and whisker plots showing scores for each variable (showing median and IQR). *n* = 5, Mann–Whitney test

The kappa score was 0.81 (95% confidence interval, 0.71–0.91), which is considered excellent. In terms of the overall graft maturation score, the BMSC group (*p* = 0.01) and PRP control (*p* = 0.03) had a significantly higher scores on average compared with the control group (Table 2) (Fig. 6).

**Table 2:** Graft Maturation Score shown as median average (IQR)

Criteria	Control (*n* = 5)	BMSC (*n* = 5)	PRP (*n* = 5)
Graft integrity	2.0 (1.5–2.0)	2.0 (2.0–2.0)	2.0 (1.5–2.0)
Synovial coverage and vascularisation	1.0 (1.0–2.0)	2.0 (2.0–2.8)	2.5 (1.8–2.8)
Graft thickness and apparent tension	0.5 (0.0–1.0)	1.0 (1.0–2.0)	2.0 (1.0–2.0)
Sealing at bone tunnel apertures	1.0 (0.0–1.0)	2.0 (1.5–2.0)	1.0 (1.0–1.5)
Overall Graft Maturation Score	5.0 (3.0–5.3)	7.0 (7.0–8.3)	6.5 (5.8–8.3)

**Fig. 7:**
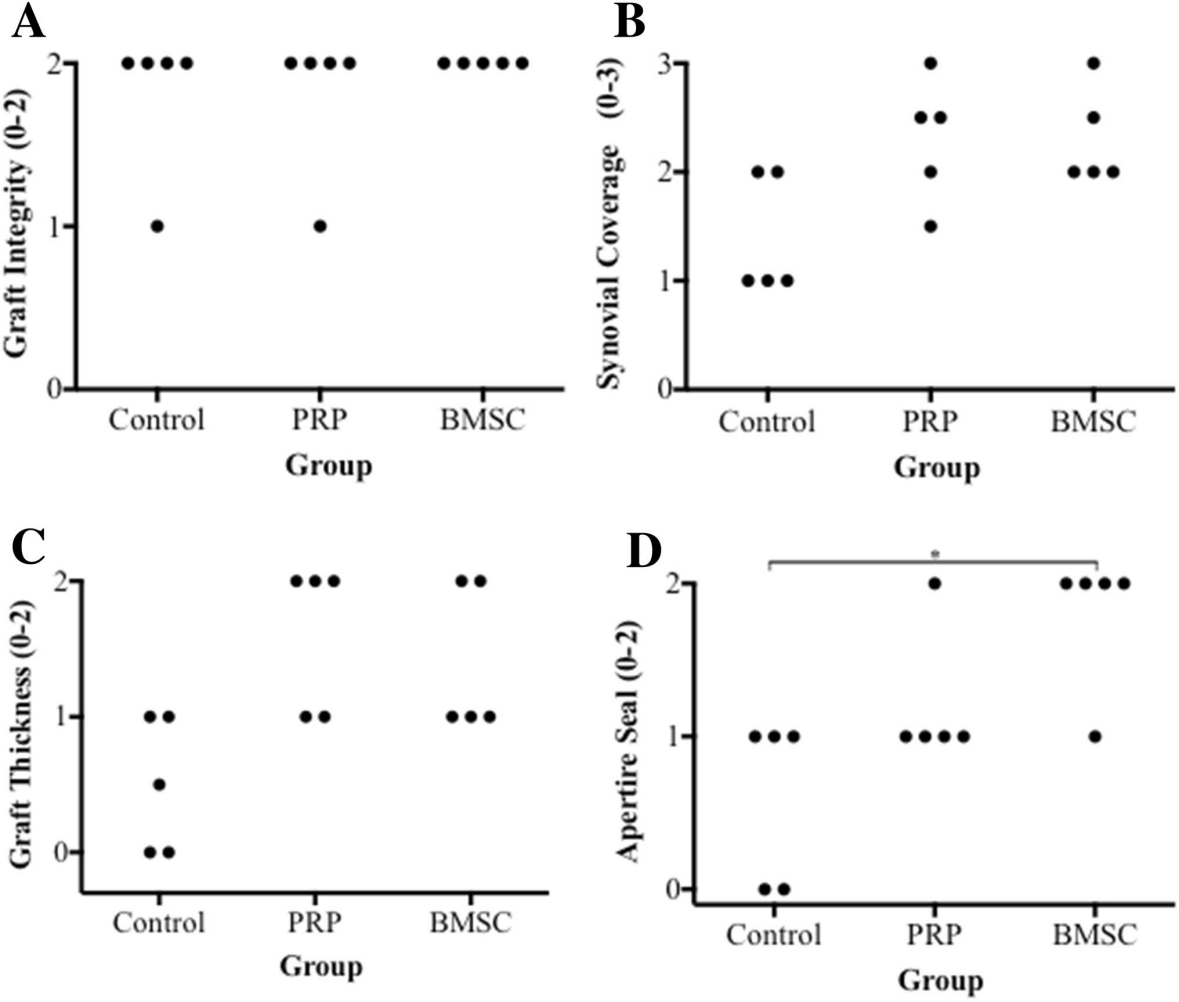
Dot plots showing scores for each variable. **a** Graft integrity. **b** Synovial coverage and vascularisation. **c** Graft thickness and apparent length. **d** Incorporation at tunnel apertures. *n* = 5, Mann–Whitney test

In terms of synovial sealing at tunnel apertures, only the BMSC group had a significantly higher score on average than the control group (*p* = 0.03) (Fig. 7d). No statistically significant differences were seen between the PRP and BMSC groups for the individual variables and total maturation scores.

### MRI assessment

#### Intra-articular graft

Graft maturation was higher in BMSC and PRP groups as evidenced by MRI maturation score and SNQ. The average graft diameters in the BMSC and PRP groups were higher than the control group. The intra-articular graft demonstrated higher signal intensity in the control group compared to the PRP and BMSC group.

### Tendon–bone healing

Tendon–bone healing next to the screws was superior to healing at the apertures in all cases in the femur. In the tibia, tendon–bone healing next to the screws was superior to healing at the apertures in all cases except the PRP group where healing was the same at the aperture and adjacent to the screw.

### Tunnel widening

The apertures in the control group were wider than seen in the other two experimental groups, and were filled with hyperintense material (Fig. 3d) (Table 3).

**Table 3:** Tunnel widening data (mm)

Tunnel segment	Control (*n* = 2)	BMSC (*n* = 2)	PRP (*n* = 2)
Femoral aperture	3.7 (3.2–4.2)	2.7 (2.2–3.2)	2.7 (2.2–3.2)
Femoral mid-portion	1.7 (1.2–2.2)	2.7 (2.2–3.2)	1.7 (1.2–2.2)
Femoral exit	3.2 (3.2–3.2)	1.7 (1.2–2.2)	2.7 (2.2–3.2)
Tibial aperture	3.7 (2.2–5.2)	1.7 (1.2–2.2)	1.7 (1.2–2.2)
Tibial mid-portion	3.2 (2.2–4.2)	2.2 (2.2–2.2)	2.2 (2.2–2.2
Tibial exit	1.7 (1.2–2.2)	2.2 (2.2–2.2	1.2 (1.2–1.2)

### Correlation between autopsy and MRI graft maturations score

The autopsy graft maturation scores and the MRI graft maturation score were significantly inversely correlated (*r* = − 0.83, *p* ≤ 0.01).

### Correlation between SNQ and Tunnel Segment Diameter.

The SNQ was positively correlated with the femoral bone tunnel diameter at the aperture (*r* = 0.88, *p* = 0.03) and mid-portion (*r* = 0.94, *p* = 0.02). No significant correlations were seen between the tibial tunnel diameter and SNQ (Table 4).

**Table 4:** Correlation analysis of tunnel diameter and signal noise quotient

Diameter of tunnel segment (mm)	Signal–noise quotient (SNQ)		Graft Maturation Score (0–9)	
	*r* value	*p* value	*r* value	*p* value
Tibia aperture	0.34	0.50	− 0.02	0.98
Tibia mid-portion	0.74	0.13	− 0.22	0.60
Tibia exit	0.50	0.21	− 0.36	0.39
Femoral aperture	0.88	0.03*	− 0.70	0.08
Femoral midportion	0.94	0.02*	− 0.65	0.12
Femoral exit	0.44	0.40	− 0.34	0.20

*p  <  0.05

## Discussion

Our first hypothesis that BMSCs and PRP would enhance graft maturation after 12 weeks was verified through significant improvements in autopsy graft maturation scores. Our second hypothesis that graft maturation at autopsy would correlate with graft maturation measured on magnetic resonance imaging (MRI) was also verified.

BMSCs have potential to modulate graft healing because of their capacity for multi-lineage differentiation and diverse paracrine effects [6]. This study supports evidence from small animals that BMSCs can enhance ACL graft healing [14, 22]. The manner by which BMSCs exert their effect on graft healing remains unknown, and future studies that tracks the cells after implantation will help reveal the mechanism. The decision to use allogeneic cells in this study rather than autologous cells was because allogeneic therapy is a more scalable treatment option due to lower processing costs [18]. A disadvantage of allogeneic cells is a higher risk of immune rejection but in this study no signs of adverse inflammatory effects were seen in this study, which is an important finding. Tunnel widening at the aperture [27] is common and is associated with synovial fluid influx [23]. An important finding of our study was that BMSCs were associated with higher level of sealing at the tunnel apertures, which might in turn reduce synovial fluid influx.

The effect of local PRP application from coated sutures has been reported in sheep [28] but to our knowledge no study has examined the effect of intra-tendinous and intra-osseous injection of PRP on tendon allograft healing. In this study, PRP treatment led to significantly enhanced graft maturation of allografts at autopsy and MRI. Nevertheless, currently there is no level 1 evidence that shows a benefit of PRP in ACL reconstruction [2]. One reason for this could be the wide variation in the PRP administration techniques because some studies inject PRP just into the graft [19], whereas others inject into the bone tunnels. To maximise exposure of the tendon–bone interface to the PRP, we recommend both intra-osseous and intra-tendinous injection [20].

MRI is a popular technique to evaluate ACL graft maturity after surgical reconstruction [26]. We demonstrated that MRI is a useful tool to measure biological modulation of graft healing because autopsy graft maturation scores correlated with MRI graft maturity scores. Zhang et al. [31] showed for the first time that graft signal intensity correlates with radius of the femoral tunnel aperture. This study corroborate these findings because we observed that SNQ correlated with the diameter of the femoral tunnel aperture and mid-portion. There is growing evidence that maturation of the intra-articular graft is related to tunnel widening in the femoral tunnel.

This study has several limitations. First, graft healing is a complex process that occurs over many months [29] but our analysis was at a single time point. Second, the number of animals included in the study was small, especially for the MRI analysis. Nevertheless, the purpose of this proof-of-concept study was to determine if MRI can detect biological modulation of graft healing and future MRI studies are now required with larger sample sizes that permit statistical comparisons. Third, histological analysis was not reported and therefore correlation of histological findings with MRI is an important area of future research. Fourth, the joint stability was not quantified using a rolimeter and biomechanical testing was not performed. Finally, fibrin sealant alone might have a positive effect by sealing the aperture at the time of surgery [23] and a better study design would have included an additional group that received fibrin sealant alone.

## Conclusion

BMSCs and PRP significantly enhanced tendon allograft maturation after 12 weeks in a translational large animal model compared to an untreated control. MRI could be used to non-invasively examine biological augmentation of ACL graft healing in clinical practice.
